# Baseline Body Composition in Prepubertal Short Stature Children with Severe and Moderate Growth Hormone Deficiency 

**DOI:** 10.1155/2016/4563721

**Published:** 2016-08-30

**Authors:** Pawel Matusik, Marta Klesiewicz, Karolina Klos, Martyna Stasiulewicz, Aleksandra Barylak, Patrycja Nazarkiewicz, Ewa Malecka-Tendera

**Affiliations:** ^1^School of Medicine in Katowice, Department of Pediatrics and Pediatric Endocrinology, Medical University of Silesia, Katowice, Poland; ^2^Scientific Society of Medical Students, Medical University of Silesia, Katowice, Poland

## Abstract

*Objective.* To compare body composition parameters in short children with severe versus moderate and no growth hormone deficiency (GHD).* Design and Method.* 61 children (40 boys) were studied. Height SDS, BMI *Z*-score, waist/height ratio (W/HtR), and body composition parameters (BIA) as fat tissue (FAT%), fat-free mass (FFM%), predicted muscle mass (PMM%), and total body water (TBW%) were evaluated. GH secretion in the overnight profile and two stimulation tests and insulin-like growth factor 1 (IGF-1) level were measured.* Results.* Overall, in 16 (26%) moderate (7.0 > peak GH < 10 ng/mL) and in 11 (18%) severe (GH ≤ 7.0 ng/mL) GHD was diagnosed. In children with sGHD BMI *Z*-score, W/HtR and FAT% were significantly higher, while FFM%, PMM%, and TBW% were significantly lower versus mGHD and versus noGHD subgroups. No significant differences between mGHD and noGHD were found. There were no differences in height SDS and IGF-1 SDS between evaluated subgroups. Night GH peak level correlated significantly with FAT%, FFM%, PMM%, and TBW%, (*p* < 0.05) in the entire group.* Conclusions.* Only sGHD is associated with significant impairment of body composition. Body composition analysis may be a useful tool in distinguishing between its severe and moderate form of GHD.

## 1. Introduction

The prevalence of growth hormone deficiency (GHD) is estimated on 1 : 4 000 to 1 : 20 000 of children which places this pathology in the group of rare diseases [1]. However, the number of children referred to Pediatric Endocrinology Clinics for short stature evaluation is much higher. Moreover, in many of them GHD based on the results of GH stimulation tests is diagnosed and they are treated for several years with daily injections of recombinant GH (rGH). Although in children with mid-line defects or in severe idiopathic GHD deficiency the diagnosis is clear and substitution of the missing hormone is essential for linear growth, glucose homeostasis, and development of normal body composition [2–4], in many short stature patients with mild GH deficiency the diagnosis remains equivocal. In this group the results of the prolonged, expensive, and invasive treatment with rGH are often not satisfactory and the individual response varies widely [5–7].

The diagnosis of GHD is based on auxological parameters and on the results of two stimulation tests which are supposed to examine the pituitary GH reserve. Various substances are being used for laboratory testing (e.g., insulin, arginine, glucagon, and clonidine) as well as overnight GH secretion, none of them being the ideal one. The tests are poorly reproducible and children with GHD quite often have a normal response in later life [8–11]. Cut-off value of peak GH secretion is also more or less arbitrary. In most pediatric endocrinology centers the diagnosis of GHD is made on the basis of peak GH concentration ≤10 ng/mL, as published in the GH Research Society guidelines in 2000 [12]. This cut-off value is being questioned by several authors who suggest that this concentration is too high and should be lowered to 7 or 8 ng/mL [9, 13, 14]. However traditionally a peak GH between 7 and 10 ng/mL is considered indicative of moderate GHD (mGHD) and children with this diagnosis are treated in the same manner as these with severe GHD (sGHD) [6, 7]. Moreover, in many mGHD patients low insulin-like growth factor (IGF-1) concentration is found, which strengthens the hypothesis that such children have clinical characteristics intermediate between sGHD and no growth hormone deficient (noGHD) short children [6].

Severe GHD induces abnormalities in body composition such as increased fat mass and reduced lean body mass [15–17]. Khadilkar et al. [18] demonstrated that one year of rGH therapy not only significantly reduced the fat mass (by 15%) but also had a beneficial effect on the cardiovascular risk reducing carotid intima media thickness (cIMT) in children with GHD. Ciresi et al. [19] showed a beneficial effect of rGH administration on metabolic parameters such as leptin level and insulin resistance (HOMA-IR) in GH deficient children. The link between rGH therapy and body composition improvement was particularly observed in children with Prader-Willi syndrome. rGH administration in these patients resulted in fat mass reduction and gain in lean body mass and was maintained over the years [20].

Body composition parameters have not been extensively studied in children with impaired GD secretion who do not fulfil the stringent criteria of sGHD and in whom mGHD is diagnosed. The aim of our study was to compare body composition parameters in short children with sGHD, mGHD, and noGHD. We hypothesized that children with mGHD will have characteristics intermediate between two other groups.

## 2. Design and Methods

### 2.1. Study Population

Study Group (SG) comprised 61 prepubertal short children (40 boys and 21 girls) at the mean age of 10.7 ± 2.6 years. They were consecutively recruited for the study from the patients referred to our Department between September 2013 and March 2014. Exclusion criteria comprised chromosomal aberrations, dysmorphic syndromes, bone dysplasia, children born small for gestational age or with intrauterine growth retardation, and patients with chronic diseases, acquired GHD, including posttraumatic or postneoplastic deficiencies.

### 2.2. GHD Diagnosis

Short stature was defined as the height below 2 standard deviation scores (SDS) from the population mean, referred to the national growth charts [21]. Peak GH concentration below 10 ng/mL in the night profile and two stimulation tests (after clonidine and insulin administration) were considered diagnostic for GHD. Stimulation test methodology was as follows: oral administration of clonidine at the dose of 0.15 mg/m^2^ body surface (Iporel, Polfa) and intravenous administration of insulin in the dose of 0.1 U/kg (Actrapid, NovoNordisk) resulting in hypoglycaemic state (serum glucose concentration below 40 mg/dL). Blood samples for GH estimation were collected every 30 minutes (from 0 to 120 min) for both tests, clonidine or insulin. GH concentrations were measured by a two-site chemiluminescent enzyme immunometric assay (hGH IMMULITE, DPC). Children with GHD were then categorized according to the maximal GH level into moderate and severe GHD (max GH ≥ 7.0 ng/mL and <7.0 ng/mL, resp.). Additionally, the concentration of IGF-1 was measured by solid-phase enzyme-labelled chemiluminescent immunometric assays (IMMULITE, DPC) in all children and was analyzed as a number of standard deviation score (SDS) from the mean. Bone age (BA) was assessed using the Greulich and Pyle method [22]. Based on the normal picture of the magnetic resonance imagining (MRI) idiopathic form of GHD was diagnosed in both mGHD and sGHD subgroups.

### 2.3. Ethical Considerations

The study was approved by the Ethics Committee of Medical University of Silesia. All parents or caregivers gave their informed consent. Patient rights were also respected according to the Declaration of Helsinki with its subsequent modifications.

### 2.4. Anthropometric Measurements

Standing height was measured by a wall-mounted Harpenden Stadiometer to the nearest 0.1 cm and weight (in patients in their underwear) by electronic scale with readings accurate to 0.1 kg. Height standard deviation score (HSDS) was calculated for all studied children referred to the national growth charts [21]. Midparental height (MPH) SDS and Δ height SDS (HSDS – MPH SDS) were also assessed. Body mass index (BMI) was calculated, using the standard formula (kilograms per meter squared). BMI *Z*-scores were derived using WHO AnthroPlus, version 1.0.4 (based on World Health Organization growth references) [23]. Waist circumference was measured midway between the lower rib margin and the iliac crest in the standing position and waist/height ratio (W/HtR) was calculated.

### 2.5. Body Composition Analysis

Body composition parameters: fat mass (FAT), fat-free mass (FFM), predicted muscle mass (PMM), and total body water (TBW) were assessed (as percentage of body weight [%]) based on bioelectrical impedance analysis (BIA) using segmental body composition analyzer (MC-980 MA Tanita Europe BV, Hoofddorp, the Netherlands).

### 2.6. Statistical Analysis

Normal distribution of all variables was confirmed by the Kolmogorow-Smirnov test. Comparative analysis of the groups for variables of normal distribution was carried out using one-way ANOVA and results were reported as least squares mean ± 95% confidence interval (CI). Further post hoc analysis was based on the Bonferroni test. Correlations between variables within studied group were based on linear Pearson's correlation coefficient. All statistical analysis was made by the Statistica*™* 10 PL software and *p* < 0.05 was considered significant.

## 3. Results

In 34 (66%) of children GHD was excluded (noGHD group). Based on the peak GH concentration 27 children with GHD were subdivided into two groups, moderate (mGHD, *n* = 16) and severe growth hormone deficient (sGHD, *n* = 11).

None of the three subgroups differed significantly by means of chronological age, BA and gender distribution (Table 1). Post hoc analysis revealed that patient with sGHD differed significantly from both mGHD and noGHD, having higher BMI *Z*-score, W/HtR, and fat tissue (FAT%) (*p* < 0.05; *p* < 0.01; *p* < 0.01, resp.), while other body composition parameters such as FFM%, PMM%, and TBW% were significantly lower (*p* < 0.01) in the sGHD group. All measured anthropometrical parameters in children with mGHD were not significantly different from children with normal GH. No statistically significant differences in height SDS, Δ height SDS, and IGF-1 SDS were found between all three evaluated subgroups (Table 1). Moreover, children with IGF-1 SDS < −2 (*n* = 20) did not differ significantly in anthropometrical and body composition parameters from those with IGF-1 SDS ≥ −2 (*n* = 41). However, peak GH level in the night profile correlated significantly with body composition parameters in the whole studied population (*r* = −0.311 for FAT%, *r* = 0.314 for FFM%, *r* = 0.313 for PMM%, and *r* = 0.313 for TBW%, *p* < 0.05) (Table 2).

## 4. Discussion

In our study we evaluated the usefulness of baseline body composition characteristics as the potential tool for confirming the diagnosis of severe or moderate GHD in short statured children. Similarly to other authors, we found an impairment of body composition in sGHD children who experienced increased fat mass and reduced lean body mass [15–17]. Children with sGHD also had significantly higher BMI *Z*-score and W/HtR. Moreover, children with sGHD have similar BA delay and no statistically different IGF-1 SDS level from those with mGHD and noGHD. Therefore in our study body composition parameters were the only ones differentiating children with sGHD from noGHDt and mGHD patients.

On the other hand we did not find any statistically significant differences neither in body composition parameters nor in BMI *Z*-score and W/HtR between mGHD and noGHD children. Therefore the study results did not show that children with mGHD had body composition impairment and did not support the hypothesis that children with GH level between 7 and 10 ng/mL have body composition characteristics intermediate between sGHD and noGHD patients.

The diagnosis of mGHD was questioned by several authors [9, 13]. Many of them believe that cut-off level of GH secretion in stimulation tests should be decreased to 7 ng/mL [9, 10, 13, 14, 24]. Wagner et al. [13] established recently a new cut-off limit of 7.09 ng/mL which was based on clinical evidence. These new cut-off limits are recommended by Murray et al. [24] in their very recent critical review on diagnosis and management of GHD in children. The need for lowering of peak GH level was also recently confirmed by Guzzetti et al. [14]. They found that the optimal GH cut-off level varies from 5.1 ng/mL (for insulin tolerance test) to 6.8 ng/mL (for clonidine). Some studies recommend sex-steroid priming before performing GH stimulation tests in prepubertal short children to confirm the diagnosis of GH deficiency [9]. However in the study by Nwosu et al. [25] hypothesis of mGHD was not supported event in group of children receiving ethinyl estradiol or testosterone enanthate priming for 5–10 days before testing. In one of our studies final height of children with mGHD treated with rGH was not significantly different from untreated peers with idiopathic short stature matched for basal age, height, and BA delay. All of them had normal GH secretion in stimulation tests repeated after cessation of therapy [8].

Results of several studies showed that majority of adolescents having received rGH treatment during childhood for GHD have normal GH secretion at retesting after growth completion [8–11]. However according to Tauber et al. [26] about 15% of them continue to have mGHD and exhibit changes in body composition after therapy cessation similar to those seen in children with sGHD, although less marked. Discontinuation of lean body mass accrual was described by Carroll et al. [27] in patients who did not continue rGH therapy after attainment of final height, but all the subjects studied had organic GHD or sGHD. Beneficial effects of rGH therapy on body fat decrease in children born small for gestational age were reported by Willemsen et al. [28] but no such effects were found by Högler et al. [29] in children with idiopathic short stature. In the available literature we did not find data comparing body composition differences in children with moderate and severe GHD treated with rGH.

In our study, significant correlations between body composition parameters and peak GH in the night profile were found. Perotti et al. [30] demonstrated that peak GH in the stimulation test (GHRH-arginine) was strongly influenced by body composition. Fat mass index alone was responsible for 34.5% of the variability in peak GH.

Significant correlation between baseline body composition assessed by BIA method and growth response to rGH therapy was found by Esen et al. [15] in children with GHD. Good responders had lower percentage of FFM and TBW compared to poor responders. Increased BMI *Z*-score and higher WHtR were predictors of more pronounced growth response in prepubertal subjects. The authors concluded that baseline body composition data can be used for prediction of growth response to rGH treatment.

Our study has two major limitations. One is the relatively small number of patients. The other is the method used for estimation of body composition by the BIA. It is not considered a gold stand technique as it may be influenced by many factors [31]. However BIA is relatively simple and noninvasive and feasible in clinical setting. Moreover, use of the dual-energy X-ray absorptiometry (DXA), which is a gold standard for the body composition analysis, is limited in children by radiation exposure, costs, and low availability. The process of BIA validation resulted in the development of standards and centile charts for healthy children [32]. Good correlation was found between BIA and dual-energy X-ray absorptiometry in the study of de Lorenzo et al. [33], Loveday et al. [34], and Kehoe et al. [35]. Moreover, our recent study confirmed its utility in children and adolescents with idiopathic scoliosis [36].

The results of our study confirm previous findings that severe growth hormone deficiency is associated with an increase in fat tissue, decrease in fat-free mass, predicted muscle mass, and total body water. In contrast our results indicate that mGHD has no significant influence on body composition in the short statured children. Therefore, body composition assessment based on BIA seems to be a useful tool in diagnosing GH deficiency in children as well as in distinguishing its severe and moderate form. However the data need to be confirmed in a prospective study based on more accurate body composition assessment methods (e.g., DXA) and on a larger group of patients.

## Figures and Tables

**Table 1:** Clinical characteristics and anthropometric measurements of studied groups.

	All patients (mean ± SD) (*n* = 61 M : F = 40 : 21)	One-way ANOVA (least squares mean ± 95 CI)				Bonferroni test
		noGHD [1] (*n* = 34 M : F = 22 : 12)	Moderate GHD [2] (*n* = 16 M : F = 9 : 7)	Severe GHD [3] (*n* = 11 M : F = 9 : 2)	*p* value	Post hoc analysis
Age (years)	10.67 ± 2.63	11.18 ± 2.66	9.91 ± 2.49	10.21 ± 2.59	NS	NS

Bone age (years)	8.18 ± 2.83	8.22 ± 3.14	7.89 ± 2.66	8.45 ± 2.41	NS	NS

Height (SDS)	−2.78 ± 0.63	−2.88 ± 0.2	−2.8 ± 0.3	−2.44 ± 0.37	NS	NS

MPH (SDS)	−0.86 ± 0.59	−0.93 ± 0.66	−0.69 ± 0.41	−0.95 ± 0.59	NS	NS

Δ height SDS	−1.84 ± 0.82	−1.81 ± 0.85	−2.11 ± 0.69	−1.48 ± 0.83	NS	NS

IGF-1 (SDS)	−1.55 ± 0.68	−1.51 ± 0.24	−1.68 ± 0.35	−1.48 ± 0.42	NS	NS

IGF-1 (ng/mL)	179.90 ± 125.73	197.54 ± 45.13	139.72 ± 62.82	167.45 ± 75.76	NS	NS

BMI *Z*-score (SDS)	−0.74 ± 1.4	−1.01 ± 0.36	−0.94 ± 0.85	0.38 ± 1.01	<0.05	[1] versus [2] *p* = NS [1]** versus **[3]** p < 0.05** [2]** versus **[3]** p < 0.05**

W/HtR	0.45 ± 0.05	0.44 ± 0.02	0.45 ± 0.02	0.49 ± 0.03	<0.01	[1] versus [2] *p* = NS [1]** versus **[3]** p < 0.01** [2]** versus **[3]** p < 0.05**

FAT (%)	20.12 ± 5.78	18.84 ± 1.82	19.45 ± 2.64	25.03 ± 3.24	<0.01	[1] versus [2] *p* = NS [1]** versus **[3]** p < 0.01** [2]** versus **[3]** p < 0.05**

FFM (%)	79.9 ± 5.78	81.17 ± 1.78	80.57 ± 2.64	74.98 ± 3.23	<0.01	[1] versus [2] *p* = NS [1]** versus **[3]** p < 0.01** [2]** versus **[3]** p < 0.05**

PMM (%)	75.51 ± 5.45	76.62 ± 1.73	76.35 ± 2.45	70.81 ± 3.06	<0.01	[1] versus [2] *p* = NS [1]** versus **[3]** p < 0.01** [2]** versus **[3]** p < 0.05**

TBW (%)	58.48 ± 4.23	59.42 ± 1.33	58.99 ± 1.98	54.84 ± 2.37	<0.01	[1] versus [2] *p* = NS [1]** versus **[3]** p < 0.05** [2]** versus **[3]** p < 0.05**

MPH: midparental height, BMI: body mass index, GHD: growth hormone deficiency, IGF-1: insulin-like growth factor 1, FAT: fat mass, FFM: fat free mass, PMM: predicted muscle mass, TBW: total body water, and W/HtR: waist height ratio. Significant differences are presented in bold.

**Table 2:** Significant Pearson's correlations between peak GH level in the night profile and body composition parameters in the study group.

All patients (*n* = 61)		
Peak GH level in the night profile		
	Pearson's correlation	Significance
FAT (%)	*r* = −0.311	*p* < 0.05
FFM (%)	*r* = 0.314	*p* < 0.05
PMM (%)	*r* = 0.313	*p* < 0.05
TBW (%)	*r* = 0.313	*p* < 0.05

FAT: fat mass, FFM: fat free mass, GH: growth hormone, PMM: predicted muscle mass, and TBW: total body water.
